# Optimal guessing in ‘Guess Who’

**DOI:** 10.1371/journal.pone.0247361

**Published:** 2021-03-10

**Authors:** Ben O’Neill

**Affiliations:** College of Science and Medicine, Australian National University, Canberra ACT, Australia; Universidad Loyola Andalucia Cordoba, SPAIN

## Abstract

Are you Richard? Are you Anne? We look at the strategic problem in the children’s guessing game *Guess Who*, which is a form of zero-sum symmetric game with perfect information. We discuss some preliminary strategic insights and formally derive an optimal strategy and win-probabilities for the game. We discuss the first-mover advantage in the game and other strategic aspects coming out of the optimal strategy. While the paper is based on the popular children’s game, our analysis generalises the actual game by allowing any initial game state with an arbitrarily large number of starting characters. With the aid of these mathematical results you can now comprehensively thrash your young children and be a terrible parent!

## Introduction

Most readers will already be familiar with the children’s game *Guess Who* which forms the basis for this article, either through having played the game during childhood, or having had children who have done so, or perhaps both. The game was designed by Theora Designs and was first manufactured by Milton Bradley in 1979. At the time of publication of this paper it is presently manufactured and distributed by Hasbro. The game has been redesigned several times and re-released in many forms. The structure of the game has remained the same since its initial design and has been a staple in toy stores for over forty years.

*Guess Who* is a two-player guessing game teaching children rudimentary skills in logic. For readers unfamiliar with the game, we set out a brief explanation. Each player has an identical game-board showing the faces of twenty-four named characters. These characters have various different characteristics—some are men, some are women, some have facial hair, some are wearing hats, some have glasses, and so on. (Some versions of the game have characters that are exclusively white, and other versions have characters that differ by race.) Each player randomly selects a character from a set of cards that matches the characters on the game-boards. Each player has a different character from the game-board and keeps this character secret from the other player. Taking it in turns, each player asks a question about the other player’s character that can be answered with a simple yes or no—e.g., “Are you wearing glasses?” (Usually the questions are framed as if each player *is* their character. Hence the language “Are you…”.) After receiving a yes or no answer the player asking the question is then able to eliminate incompatible characters on his game-board by placing these face down, so that only the remaining face-up characters are possible characters that might be held by the other player. By guessing the characteristics of characters, player narrows down the search through this process of elimination. When one player is able to correctly guess the other player’s character as his question, he wins the game; specific variations in rules for the final guess are discussed soon.

For many young children, *Guess Who* it is their first exposure to the basic logical idea of eliminating a false hypothesis through contradictory information. As children progress in their understanding of the game they can also learn some basic probabilistic intuition which gives them a higher chance of winning the game. These features make the game a good introduction to logical reasoning. As an object of academic study, the game is useful because it is a simple discrete “search race” that bears resemblances to more complex problems in sports, industrial competition, and other competitive ventures. In this regard, the game has two interesting strategic aspects that occur in broader competitive problems: (1) the agents are able to choose between more “conservative” and more “risky” strategies in their moves; and (2) the agents are able to observe the progress of their competitor and adjust their own strategy accordingly.

Formally, *Guess Who* is a “search race” in which each player is searching for an unknown item which is known to be equiprobable from a finite group of objects. Search algorithms for binary search are studied extensively in computer science and have led to the use of the “split-in-half search” which operates by splitting the remaining objects evenly (or as close as possible if there are an odd number of objects) on each search iteration (see e.g., [1–3]). (Much of the literature in this area uses the term ‘binary search’ to refer to the split-in-half-search. This is an unfortunate terminology, since it conflates an optimal search procedure within a binary choice setting with the choice setting itself. To avoid this conflation of issues, we instead use the term ‘binary search’ to refer to any search done within the context of a set of items with a single search-for item.) This same method is referred to as the “split-half heuristic” in psychology and information theory (see e.g., [4, 5]). This search method maximises the expected number of objects eliminated in each guess (equivalently, the expected information gain), and thereby minimises the expected number of guesses taken to complete the search. In a regular search problem without an opponent it is commonly used as the most efficient search method. However, in a search race the situation is complicated by the fact that the goal is to win the race, rather than minimising the expected number of guesses. Intuitively, we would expect that the efficient split-in-half search would be worthwhile for the player who is ahead in the race, but might be suboptimal for the player who is behind, since he must take a risk at some point in the race to try to overtake his opponent. We therefore expect the optimal strategy to be a mixture of split-in-half search, and some “risky play” which seeks to overcome a disadvantage in the game state.

*Guess Who* is advertised as being suitable for children aged six and up, but in the experience of the present author it is also suitable (and fun) for younger children—essentially any child who is old enough to ask and answer basic yes-no visual questions about faces. The game is useful in teaching young children rudimentary logic and efficient searching. Psychological research has analysed the types of questions asked by people of different age groups in this kind of search game and in similar search games (see e.g., [4, 6–11]). This research has found that young children tend to ask about a specific object (e.g., “Are you John?”) whereas older children ask more efficient questions to eliminate more characters (e.g., “Are you wearing glasses?”). Young children also ask ‘pseudo-specific’ questions, such as asking “Do you have a beard?” when only one of the remaining characters has a beard. This suggests a focus on individual objects by young children. Nelson *et al*. [12] find that ten-year olds have some ability to use efficient searches based on observed characteristics, and they are able to adapt their search method to changing characteristics to some degree, partially preserving efficient searching as characteristics are exogenously changed.

## Defining the strategic problem in two variations of Guess Who

In this paper we will consider a generalised variant of *Guess Who* with a game-board that has an arbitrary number of characters. We will look at the strategy of this game starting from any given game-state, which is a specification of the number of remaining characters on the game-board of each player. We assume that the only information available to each player is the state of their own game-board and the series of questions asked during the game. This is sufficient to allow each player to determine the state of the opponent’s game board other than determining which character they have eliminated due to holding that character. Each player is aware of the game-state at each point and their decision is determined entirely by this game-state. To be clear, we assume that there is no sneaky business insofar as using other information from the opponent’s behaviour or anything else which would give a strategic advantage from matters outside of the game. This assumption excludes strategies that infer opponent’s likely actions from exogenous behavioural clues.

Each player is able to ask questions that will effectively seek to confirm or eliminate a given subset of the characters remaining on his game-board. Although it is common for questions to refer to characteristics like hair colour, facial hair, etc., it is always possible to refer to any chosen subset by specification of the logical disjunction of the characters by name—e.g., “Are you Max or Claire or Anne or Tom or … or John?” It is also possible to ask a simple ranking question to refer to a subset of any chosen size—e.g., “Does your name come before the word ‘Milk’ in the alphabet?” (While strategically sound, this is not recommended when playing with young children!) Under such an approach the names and characteristics of the characters are arbitrary, and confer no useful information for the strategic decision. The strategic choice for a player is to decide *how many* characters to test with his guess.

Suppose that a player is in the game-state (*n*, *m*) where he has n∈N characters remaining on his game-board and his opponent has m∈N characters remaining, and it is his turn to guess. We let 1≤*s*(*n*, *m*)<*n* be the integer number of characters the player chooses as a subset to confirm or eliminate with his guess, disallowing the empty or full sets. It is notable that there is a partial symmetry to our problem—if the player chooses a subset of *s*>1 characters, this is equivalent to choosing the other subset of *n*−*s* characters.

Our analysis will examine two variants of the game, using different rules for the “final guess” to win the game. The official game rules provide that a player may use their move to make a guess of the opponent’s character—if the guess is correct they win the game but if the guess is wrong they lose the game (https://www.hasbro.com/common/instruct/GuessWho.PDF). Correspondingly, the player does not win the game merely by narrowing down to a single character until they make a “final guess” of this character. We will denote this “final guess” as the move *s* = 0, and we note that this move is distinct from the move *s* = 1, where the player also attempts to narrow down to a single character, but does not make this character his “final guess” (and therefore does not win or lose at the end of the move). We will also examine a rules variant where a player can guess a single character to win, but does not lose if they guess incorrectly. (This rules variant is more fun when playing with young children, since they do not lose the game so easily.) In this latter case we take the move *s* = 1 to be a winning move if it succeeds in narrowing the characters to a single remaining character (and the move *s* = 0 is not required for the analysis). In this variant the move *s* = 1 is **not** equivalent to *s* = *n*−1 since the former guess allows the player to win on the present turn while the latter does not.) These two rules variations are summarized in Table 1 below.

**Table 1:** Rules variants for analysis of *Guess Who*.

	Official Rules (I)	Variant Rules (II)
Winning and losing the game	Guess a single character—correct guess wins the game; incorrect guess loses the game	Guess a single character—correct guess wins the game; incorrect guess does not lose the game
Allowable moves	0≤*s*(*n*, *m*)<*n*	1≤*s*(*n*, *m*)<*n*
Win condition (on own turn)	*s*(*n*, *m*) = 0 (correct guess)	*s*(*n*, *m*) = 1 (correct guess)
Loss condition (on own turn)	*s*(*n*, *m*) = 0 (incorrect guess)	None
Win probability from state (*n*, *m*)	*p*_I_(*n*, *m*)	*p*_II_(*n*, *m*)

We consider the case where there is a fixed positive payoff for winning and an opposing loss for losing leading to a zero-sum game (we refer to this particular type as a “win-loss” game). Under this system of payoffs, each player seeks to maximise the probability of winning the game, and adopts a strategy with a view to this optimisation criterion. Since the probability of winning depends on the strategy of the other player, this is a game-theory problem. Ours is a two-person win-loss game with perfect information, which is presented in extensive form. Our generalised problem is a specification of the set of games corresponding to every possible starting game-state (*n*, *m*) with n∈N and m∈N. Each of these game-states is a subgame of the larger generalised “game” and each subgame starting from a specified game-state is a two-person finite win-loss game with perfect information. (Technically the larger generalised problem is not a game (in the game theoretic sense) since it does not include specification of the starting game-state (or specification of a mechanism to determine this). The generalised problem is technically a countably infinite set of games for which each game is a sub-game of some larger game in the set. If we were to specify a probabilistic mechanism for determining the starting game-state then this would become a game in the proper technical sense.) Although each of the game-states is non-symmetric, owing to the fact that there is a moving and a waiting player, the generalised infinite “game” is symmetric in the sense that the infinite set of available strategies is the same for each player and the payoff structure is symmetric for the two players.

Optimal strategy properties for two-person finite zero-sum games with perfect information are well-known (see e.g., [13]). In particular, a win-loss game has a “minmax value” which is the value that maximises the minimum win probability for each player over all possible strategies. (In general this minmax value maximises the minimum “expected payoff” of each player. In this problem the expected payoff is a positive linear function of the win probability so maximisation of the expected payoff is equivalent to maximisation of the win probability. For the present type of game the minmax theorem holds that these two values are equal for both players, and so there is a single minmax value—see e.g., Maschler, Solan and Zamir [13], Theorem 4.43, p. 115.) A strategy is “optimal” if it achieves this minimax value. Any combination of optimal strategies for the two players constitutes a Nash equilibrium, and any such equilibrium gives optimal strategies for the players. Since each subgame is also a finite win-loss game, the same result ensures that any combination of optimal strategies for the two players is a subgame-perfect Nash equilibrium. The optimal strategy for our generalised game can therefore be derived by backwards induction using a recursive method that makes use of this equilibrium criterion. Although these existence results do not guarantee an optimal strategy which is a “pure strategy” (i.e., which involves only deterministic moves) we will show that our game has a set of optimal pure strategies which are each best responses to all other optimal strategies. Since our generalised game is symmetric, the solution is also symmetric, in the sense that both players have the same set of optimal strategies. We will therefore obtain a single set $S_{*}$ of optimal strategies, where any pair of strategies from the set constitutes a subgame-perfect Nash equilibrium.

## Recursive win probabilities for two variants of Guess Who

Our strategic analysis of *Guess Who* allows any game-state (*n*, *m*) with n∈N and m∈N. Each player adopts a strategy s≡(s(n,m)|n∈N,m∈N) for the generalised game which is an array of individual strategies for each possible game-state. We consider the case where the player about to take his turn (the “moving player”) adopts strategy ***s***_1_ and the other player (the “waiting player”) adopts strategy ***s***_2_. For brevity we let *s*≡*s*_1_(*n*, *m*) denote the move chosen by the moving player in the current game state. The game rules provide that each character is selected at random; we take this as simple random sampling without replacement, so that each character is marginally uniformly distributed over all characters not already eliminated.

Under the official rules (I) the moving player can guess *s* = 0, in which case he wins if he guesses correctly (with probability 1/*n*) and loses if he guesses incorrectly. Alternatively, he can guess *s*≥1 in which case he cannot win with his present guess, but he still reduces the characters on his game-board and passes the opportunity to move to his opponent. From these options, we see that the probability that the moving player wins is given recursively by:

$$\begin{array}{l} p_{I}(n,m|s_{1},s_{2})=\left\{ \begin{array}{ll} \;\frac{1}{n} & s=0,\\ \frac{s}{n}∙(1-p(m,s|s_{2},s_{1}))+\frac{n-s}{n}∙(1-p(m,n-s|s_{2},s_{1})) & s\geq 1, \end{array}\right.\\ \;=\left\{ \begin{array}{ll} \;\frac{1}{n} & s=0,\\ 1-\frac{s}{n}∙p(m,s|s_{2},s_{1})-\frac{n-s}{n}∙p(m,n-s|s_{2},s_{1}) & s\geq 1. \end{array}\right. \end{array}$$

(Observe that in these equations the probability functions on the right-hand-side of the equation now have the strategies reversed, since the waiting player becomes the new moving player and *vice versa*.)

Under the variant rules (II) the moving player can guess *s* = 1, in which case he wins if he guesses correctly (with probability 1/*n*) but does not lose if he guesses incorrectly. (Some versions of the game rules allow a player to ask a question which would narrow the game-board down to a single character without actually guessing this character for the win—e.g., asking “Are you wearing a hat?” when there is only a single remaining character wearing a hat. Even if this is allowable, it is never a rational strategy. In such cases it is always better to take a guess of the character that would allow the player to win if correct—e.g., “are you Harold?” We assume that this is always done.) If he does not win with his present guess he reduces the number of characters on his game-board and passes the move to his opponent (who now becomes the moving player). Alternatively, he can guess *s*>1 in which case he cannot win with his present guess, but he still reduces the characters on his game-board and passes the opportunity to move to his opponent. From these options, we see that the probability that the moving player wins is given recursively by:

$$\begin{array}{l} p_{II}(n,m|s_{1},s_{2})=\left\{ \begin{array}{rr} \frac{1}{n}+\frac{n-s}{n}∙(1-p(m,n-s|s_{2},s_{1})) & s=1,\\ \frac{s}{n}∙(1-p(m,s|s_{2},s_{1}))+\frac{n-s}{n}∙(1-p(m,n-s|s_{2},s_{1})) & s>1, \end{array}\right.\\ \;=\left\{ \begin{array}{rr} 1-\frac{n-s}{n}∙p(m,n-s|s_{2},s_{1}) & s=1,\\ 1-\frac{s}{n}∙p(m,s|s_{2},s_{1})-\frac{n-s}{n}∙p(m,n-s|s_{2},s_{1}) & s>1. \end{array}\right. \end{array}$$

Since our generalised “game” is symmetric, it follows that both players must have the same set of optimal strategies, and each optimal strategy pair is a subgame-perfect Nash equilibrium. Hence, we are able to identify the optimal strategies using the recursive equations that come from this equilibrium. Let S be the set of all possible pure strategies and let $s_{*}\in S$ be an optimal pure strategy. If both players adopt this strategy this gives a subgame-perfect Nash equilibrium characterised by the following requirement:

$$p(n,m|s_{*},s_{*})=\underset{s\in S}{\max}\;p(n,m|s,s_{*})\;\mathrm{for}\;\mathrm{all}\;n,m\in N.$$

(In cases where we are concerned only with the optimal strategy for a game with a given number of starting characters, we can restrict the range of *n* and *m* accordingly. However, it is just as easy to proceed for the general case, allowing an arbitrary number of characters. This gives the optimal strategies for specific game-states as a consequence.)

The above optimisation criterion leads us to a recursive equation for the optimal strategy. Since both players are using the same strategy we drop the reference to the strategy s_*_ and we write the probability that the moving player wins simply as *p*(*n*, *m*) = *p*(*n*, *m*|s_*_, s_*_). It is also helpful to conduct our analysis in terms of *a*(*n*, *m*) = *nm*∙*p*(*n*, *m*). The optimization criterion leads to the following simple recursive equations for the two variants. Under the official rules (I) we have the recursion:

$$\begin{array}{l} \;a_{I}(n,m)=\underset{0\leq s<n}{\max}a_{II}(n,m|s),\\ a_{I}(n,m|s)=\left\{ \begin{array}{ll} \;m & s=0,\\ nm-a_{I}(m,s)-a_{I}(m,n-s) & s\geq 1, \end{array}\right. \end{array}$$

Under the variant rules (II) we have the recursion:

$$\begin{array}{l} \;a_{II}(n,m)=\underset{1\leq s<n}{\max}a_{II}(n,m|s),\\ a_{II}(n,m|s)=\left\{ \begin{array}{ll} \;nm-a_{II}(m,n-s) & s=1,\\ nm-a_{II}(m,s)-a_{II}(m,n-s) & s>1. \end{array}\right. \end{array}$$

Any pure strategy satisfying these recursive equations for all game-states is an optimal pure strategy of the infinite generalised problem. Duersch, Oechssler and Schipper [14] give some existence results for pure strategy equilibria in two-person symmetric zero-sum games. They find that these equilibria exist so long as the game avoids a certain kind of cyclical optimal response similar to what arises in the game of Rock-Paper-Scissors. In our case the existence of a pure strategy solution is guaranteed by the fact that each maximisation in the above recursive equations is done over a finite set of possible guesses and each is purely recursive based on past solutions (i.e., there is no cyclic relationship in the equations). This ensures that there will be a set of guesses for each game-state that are optimal and thereby combine to yield an optimal strategy. In fact, we will see that—for game states that are not too small—there are multiple pure strategies that are optimal, since maximisation of the win-probability in the first equation does not always give a unique optimal guess.

We can obtain the optimal strategy by solving the above recursive equations using backwards induction, starting with the case where the moving player has a single remaining character. In this case we know that the player will guess this remaining character and will be correct, thereby winning the game. For all m∈N this gives us *s*(1, *m*) = 1 with corresponding win-probability *p*(1, *m*) = 1 giving *a*(1, *m*) = *m*. (This applies under both variants of the game.)

Starting with this baseline case and using the technique of backwards induction we can obtain the win-probability function under optimal strategy. Matrices showing the resulting functions *a*_I_ and *a*_II_ for values 1≤*n*, *m*<24 (i.e., over all game states in the standard board game) are shown at the end of this paper.

## Analysis of first-mover advantage and game-state advantage

The recursive formulae in the previous section allow computation of the win-probabilities for both rules variants under optimal play. Using these win-probabilities it is possible to quantify the first-mover advantage and game state advantage that accrue to the moving player. These can be defined as the symmetric and anti-symmetric parts of the win-probability function:

$$\begin{array}{ll} \mathrm{First}‐\mathrm{mover}\;\mathrm{advantage} & F(n,m)=\frac{1}{2}(p(n,m)+p(m,n)‐1),\\ \mathrm{Game}‐\mathrm{state}\;\mathrm{advantage} & G(n,m)=\frac{1}{2}(p(n,m)‐p(m,n)), \end{array}$$

which are derived from the requirements:

$$p(n,m)=\frac{1}{2}+G(n,m)+F(n,m)\;F(n,m)=F(m,n)\;G(n,m)=-G(m,n).$$

**Fig 1:**
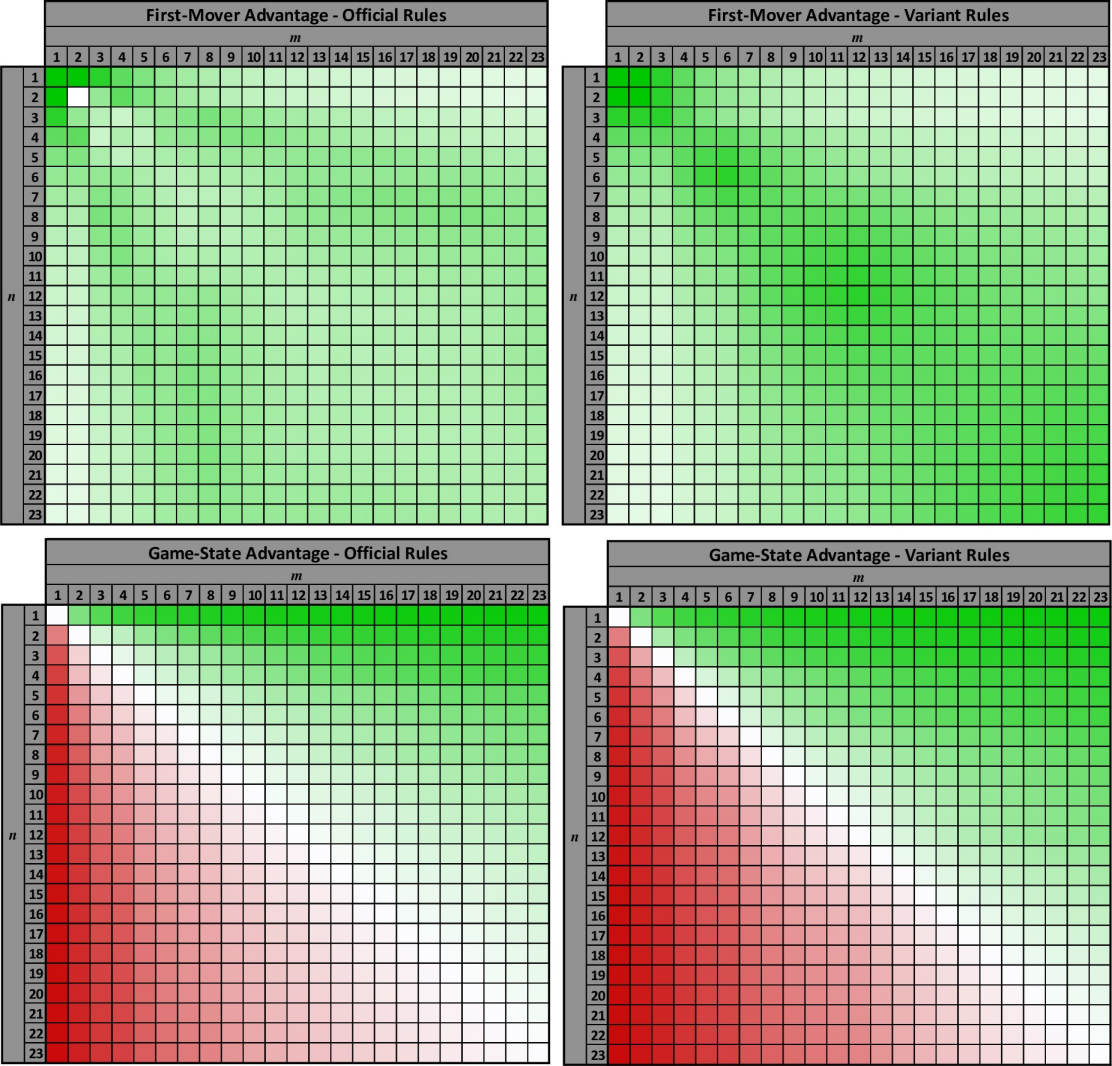
First-mover advantage and game-state advantage for both rules variants (all plots are based on optimal play by both players).

The first-mover advantage and game-state advantage under both rules variants is shown below in the heatmaps in Fig 1. Intense green on the heatmap represents a high positive value and intense red represents a high negative value. The symmetry of the first-mover advantage and anti-symmetry of the game-state advantage is clearly evident in the heatmaps.

**Fig 2:**
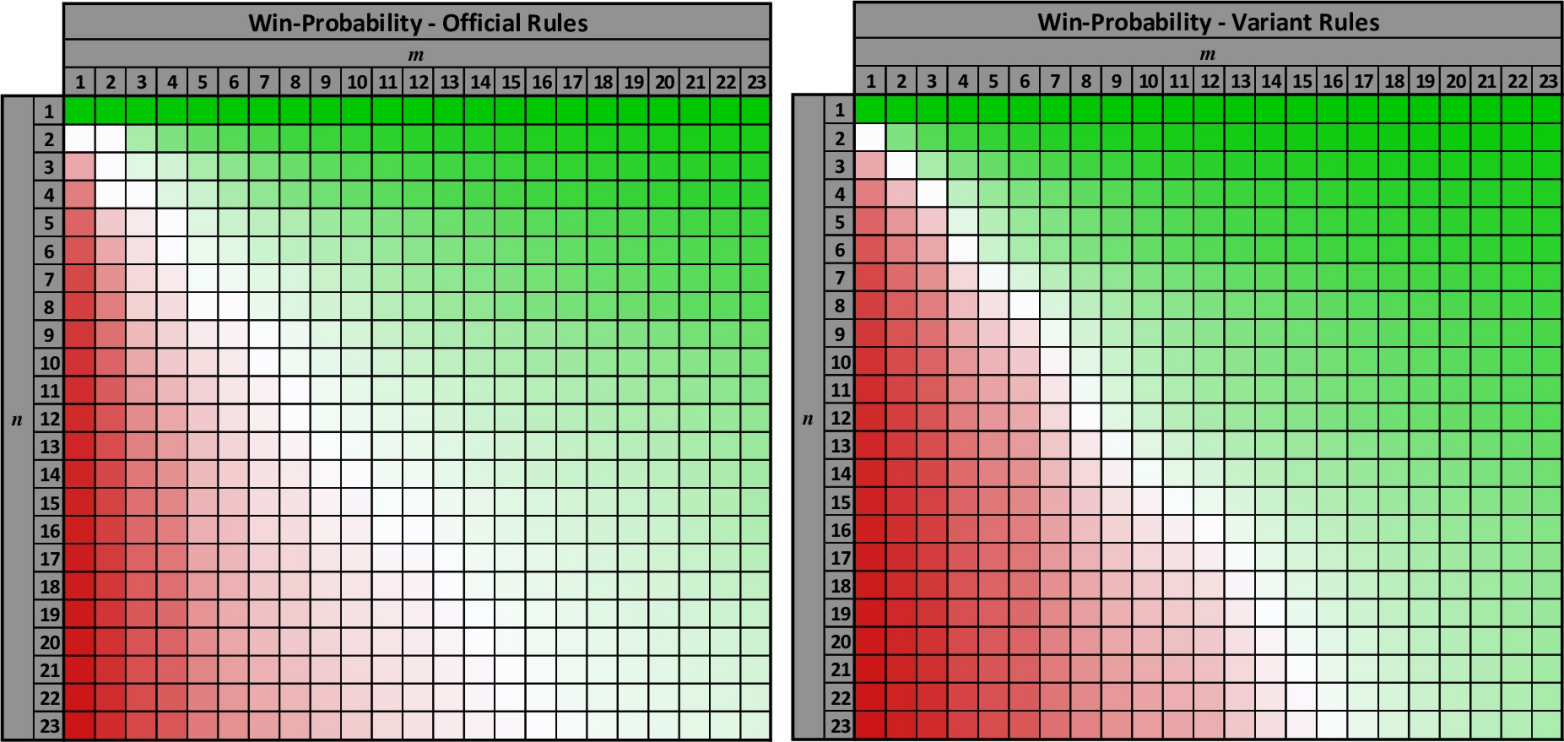
Win probabilities for both rules variants (all plots are based on optimal play by both players).

As can be seen from Fig 1, the official rules provide a smaller first-mover advantage and a smaller game-state advantage. This occurs because the special move for a “final guess” takes an additional turn beyond the turns used to (safely) narrow down the characters, and so the game is “slower” than under the variant rules. This slower game means that the first-mover advantage and the game-state advantage are ameliorated to some extent. (It is interesting to note that the rules of *Guess Who* provide that the youngest player should always move first. This rule implicitly recognises the first-mover advantage in the game and attempts to ameliorate this advantage by giving the first move to the player expected to use the most sub-optimal play.) It is also interesting to observer that, aside from cases of low values for *n* and *m*, the first-mover advantage under the official rules is higher in the bifurcating regions where the game-state is uneven, whereas the first-mover advantage under the variant rules is higher where the game-state is even. The win-probability is the combination of these two effects—it is shown in Fig 2 below. As can be seen from the heatmaps in the figure, the first-mover advantage shifts the line of fair game states away from those with no game state advantage, so that the fair states do not occur on the main diagonal of the graph.

A standard game of *Guess Who* has twenty-four characters and each player eliminates his own character from consideration before guessing. This means that the game starts at game state (23, 23) (shown as the bottom-right corner of all the heatmaps). If both players play an optimal strategy then the win probabilities for the first player under the two rules variants are:

$$\begin{array}{cc} \mathrm{Official}\;\mathrm{Rules}\;(I) & p_{I}(23,23)=\frac{296}{529}=0.5595,\\ \mathrm{Variant}\;\mathrm{Rules}\;(II) & p_{II}(23,23)=\frac{349}{529}=0.6597. \end{array}$$

Although *Guess Who* has a first-mover advantage, it is possible to correct this to create a “fair” game by altering the starting state of the game to one where there is a game-state advantage to the waiting player that is equivalent to the first-mover advantage of the moving player. The fair game-states where this occurs are shown in the Table 2 below. For example, under the official rules one can create a fair game by allowing the waiting player to eliminate seven incorrect characters from their game board prior to the start of the game, so that the game begins in game state (23, 16). This elimination of characters balances out the first-mover advantage of the moving player.

**Table 2:** Fair game states with 1≤*n*, *m*<24 (based on optimal play by both players).

**Official Rules (I)**	(2,1), (2,2), (3,2), (4,2), (4,3), (5,4), (6,4), (8,5), (10,7), (11,8), (12,8), (14,10), (16,11), (16,12), (18,13), (20,14), (22,15), (23,16)
**Variant Rules (II)**	(2,1), (3,2), (4,3), (6,4), (8,6), (12,8), (16,2)

The recursive formulae above allow easy derivation of matrices for *a*_I_(*n*, *m*) and *a*_II_(*n*, *m*) for any range 1≤*n*, *m*<*U*, which then gives the corresponding values for the win probabilities, first-mover advantage and game-state advantage, under either rules variant. In the next section we derive a non-recursive formula for the win probability and optimal pure strategies for the variant rules.

## The optimal pure strategies and their intuitive properties (variant rules only)

We will conduct an analysis of the optimal strategy only for the variant rules (II). This is the more interesting of the two cases, since it holds a closer resemblance to other “search race” game theoretic problems in broader fields such as industrial competition. It also has smoother properties for its win-probability function and a relatively simple form for its strategic optima, which give some intuitive insight into the nature of a “search race” problem.

We present our optimality findings as a series of theorems, with all proofs in the S1 Appendix. The strategic solution shown in this section was initially derived by the author using the “guess and verify” method, by first observing that the forward differences *a*_II_(*n*, *m*+1)−*a*_II_(*n*, *m*) have a simple form that can be expressed using a number series introduced in Theorem 1 below.

**Theorem** **1:** The win-probabilities when both players use optimal strategies are:

$$p_{II}(n,m)=\frac{a_{II}(n,m)}{nm}\;a_{II}(n,m)=\sum_{k=1}^{m}\min\left(n,⋆k\right),$$

where the values ⋆*k* are taken from the following series:

$${\left(⋆k\right)}_{k\in N}=(1,2,3,\underset{3\;\mathrm{times}}{\underset{︸}{6,\ldots ,6}},\underset{6\;\mathrm{times}}{\underset{︸}{12,\ldots ,12}},\underset{12\;\mathrm{times}}{\underset{︸}{24,\ldots ,24}},\underset{24\;\mathrm{times}}{\underset{︸}{48,\ldots ,48}},\ldots ).$$

**Theorem 2**: We can rewrite the function *a*_II_ in simplified form as:

$$a_{II}(n,m)=\left\{ \begin{array}{rr} nm-\frac{⋆n}{2}(n-\frac{⋆n}{3})+\frac{1}{3}∙I(n\leq 2) & \mathrm{if}\;⋆n\leq ⋆m,\\ ⋆m(m-\frac{⋆m}{3})+\frac{1}{3}∙I(m\leq 2) & \mathrm{if}\;⋆n>⋆m. \end{array}\right.$$

**Theorem** **3:** Any pure strategy s_*_ is an optimal pure strategy (under the variant rules) if and only if its guesses all fall within the range ${\underline{s}}_{*}(n,m)\leq s_{*}(n,m)\leq {\bar{s}}_{*}(n,m)$ with the following lower and upper bounds:

$$\begin{array}{ll} {\underline{s}}_{*}(n,m)=1 & \mathrm{if}\;n\leq 3,\\ {\underline{s}}_{*}(n,m)=☆n & \mathrm{if}\;n\geq 4\;\mathrm{and}\;⋆n\leq ⋆m,\\ {\underline{s}}_{*}(n,m)=\left(⋆m\right)/2 & \mathrm{if}\;n\geq 4\;\mathrm{and}\;⋆n>⋆m, \end{array}$$

$$\begin{array}{ll} {\bar{s}}_{*}(n,m)=1 & \mathrm{if}\;n\leq 3,\\ {\bar{s}}_{*}(n,m)=1 & \mathrm{if}\;n\geq 4\;\mathrm{and}\;m\leq 2,\\ {\bar{s}}_{*}(n,m)=2 & \mathrm{if}\;n=4\;\mathrm{and}\;m\geq 3,\\ {\bar{s}}_{*}(n,m)=n-{\underline{s}}_{*}(n,m) & \mathrm{if}\;n>4\;\mathrm{and}\;m\geq 3, \end{array}$$

where the values ☆*k* are taken from the following series:

$${\left(☆k\right)}_{k\in N}=(\underset{3\;\mathrm{times}}{\underset{︸}{1,\ldots ,1}},1,2,3,\underset{3\;\mathrm{times}}{\underset{︸}{3,\ldots ,3}},4,5,6,\underset{6\;\mathrm{times}}{\underset{︸}{6,\ldots ,6}},7,8,9,10,11,12,\underset{12\;\mathrm{times}}{\underset{︸}{12,\ldots ,12}},13,\ldots ).$$

**Corollary 1**: The following strategy s_*_ is an optimal pure strategy (under the variant rules):

$$s_{*}(n,m)=\left\{ \begin{array}{cc} \max\left(1,\lfloor n/2)\right\rfloor & \mathrm{if}\;m\geq 3,\\ 1 & \mathrm{if}\;m\leq 2. \end{array}\right.$$

**Corollary 2**: The following strategy s_*_ is an optimal pure strategy (under the variant rules):

$$s_{*}(n,m)=\left\{ \begin{array}{cc} \max\left(1,\lfloor n/2\rfloor \right) & \mathrm{if}\;⋆n\leq ⋆m,\\ \max\left(1,\lfloor \left(⋆m\right)/2\rfloor \right) & \mathrm{if}\;⋆n>⋆m. \end{array}\right.$$

**Remark**: Another way of expressing this solution is as follows. For simplicity, consider the case where *n*≥4 and *m*≥4. If 3∙2^*k*^<*n*≤3∙2^*k*+1^ and *m*>3∙2^*k*^ for some integer *k*≥0 then we have ⋆*n* = 3∙2^*k*+1^≤⋆*m*. In this case we have:

$$p_{II}(n,m)=1-\frac{3∙2^{k}}{m}\left(1-\frac{2^{k+1}}{n}\right).$$

Alternatively, if *n*>3∙2^*k*+1^ and 3∙2^*k*^<*m*≤3∙2^*k*+1^ for some integer *k*≥0 then we have ⋆*n*>3∙2^*k*+1^ = ⋆*m*. In this case we have:

$$p_{II}(n,m)=\frac{3∙2^{k+1}}{n}\left(1-\frac{2^{k+1}}{m}\right).$$

These theorems set out the optimal strategies for *Guess Who* under the variant rules, and the win-probabilities that result when both players use an optimal strategy. Theorems 1–2 give the win-probability from any game-state in the case where both players play an optimal strategy. Theorem 3 shows us that there is a wide scope for the optimal strategy, with a range of allowable guesses in each game-state. It is notable that each optimal strategy is in accordance with our preliminary intuition regarding the strategies for a player who is ahead or behind in the game. In particular, we can see from Corollary 1 that one optimal strategy is for the player to use the ‘split-in-half search’ as his guess, taking *s* = max(1,⌊*n*/2⌋) for all his moves unless his opponent has only one or two characters left. In this latter case the opponent is sufficiently close to winning the game that the moving player should make a guess *s* = 1 of a single character, even if this is not the split-in-half strategy from his position. This optimal strategy shows us that the player who is behind in the game may delay his ‘risky search’ until quite late, when his opponent has only one or two characters remaining on the game board.

On the other hand, it is possible to undertake risky play earlier in the game while still using an optimal strategy. In particular, we can see from Corollary 2 that another optimal strategy is for the moving player to use a ‘split-in-half search’ if he has a sufficiently small number of remaining characters relative to his opponent (i.e., when ⋆*n*≤⋆*m*). If he does not have a sufficiently small number of characters then it is optimal for the moving player to take a ‘risky search’ where he takes *s* = max(1,⌊(⋆*m*)/2⌋) as his guess. Unlike the split-in-half search, this guess does not minimize the expected number of characters eliminated, but it is equally optimal due to the fact that the moving player is sufficiently far behind in the search race to warrant a risk being taken to overtake his opponent. Even in this case we see that the risky search bears a resemblance in form to the split-in-half search—it is made based on a number of characters related to the number the opponent has remaining.

The wide class of optimal strategies shows us that there is a degree of robustness in choosing a strategy in *Guess Who*. However, the range of optimal strategies encompass the use of the ‘split-in-half search’ when the moving player has a sufficiently small number of characters, and an allowance for a ‘risky search’ at some point if the game-state becomes sufficiently dire to warrant this. The range of optimal strategies allows some deviation from this, but the optimal strategies are all quite close to this archetype.

We have considered only pure strategies in our analysis. However, it is notable that, aside from the pure strategies presented in Theorem 3, any probabilistic mixture of these optimal strategies is an optimal mixed strategy that also leads to a subgame-perfect Nash equilibrium. The set of optimal strategies includes any mixed strategy where the guess in each game-state is chosen from a distribution with support over the range of guesses shown in Theorem 3. Like the set of pure strategies, this set can also be considered as effectively being a single equilibrium since every strategy in the set is an optimal response to all strategies in the set.

It is useful to be able to look at how the win-probability varies according to the game-state, using the simplified expression in Theorem 2. This allows us to express the win-probability in a form that is more amenable to further analysis. The last term in the expression is just a slight correction term that applies in low game state values—it is not really of much interest. The more interesting case occurs when *n*≥3 and *m*≥3. In this case we can write the win-probability in terms of the quantities:

$$r=\frac{n}{m}\;d_{n}=\frac{n}{⋆n}\;d_{m}=\frac{m}{⋆m}.$$

For *n*≥3 and *m*≥3 we can use Theorems 1–2 to write the win-probability as:

$$p_{II}(n,m)=\left\{ \begin{array}{rr} 1-\frac{1}{2}∙r∙A(d_{n}) & \mathrm{if}\;r\leq d_{n}/d_{m},\\ \frac{1}{r}∙A(d_{m}) & \mathrm{if}\;r>d_{n}/d_{m}, \end{array}\right.$$

where *A*(*x*)≡(3*x*−1)/(3*x*^2^). It is easy to show that ½<*d*_*n*_≤1 and ½<*d*_*m*_≤1 so that 2/3≤*A*≤3/4 in both parts of the expression. (This last inequality is derived by maximising and minimising the function *A* over the specified range.) This is a narrow range, which means the main thing that affects the win-probability is the ratio *r*, as we expect.

The pattern of values in the win-probability function should now be a bit more discernible. In the case when *n*≥3 and *m*≥3, if the ratio *r* is held constant and the magnitude of the values of *n* and *m* are increased (in proportion to each other), the function oscillates slightly due to the changes in the function *A*, but it holds its value approximately. If the magnitude of the values of *n* and *m* is increased by an integer power of two then the win-probability remains unchanged (i.e., *p*_II_(2^*k*^*n*, 2^*k*^*m*) = *p*_II_(*n*, *m*) for all k∈N so long as *n*≥3 and *m*≥3) but if a different multiple is applied then there can be a slight change in the function, owing to the oscillation in *A* that we mentioned. Fair game-states, where each player has an equal chance of winning, occur in cases when *r* = 3/2 (*d*_*n*_ = 1) or *r* = 4/3 (*d*_*m*_ = 1). The win-probability for the game has already been shown in Fig 2 (right heatmap) and this pattern in the values is discernible from that plot.

Our present results also allow us to further elucidate the first-mover advantage and game-state advantage for the variant rules shown in Fig 1 (right heatmaps). The first-mover advantage under the variant rules accrues most intensely at the points where *n* = ⋆*n* = ⋆*m* = *m* (i.e., where *r* = *d*_*n*_ = *d*_*m*_ = 1). At these points we have *G*_II_(*n*, *m*) = 0 and *F*_II_(*n*, *m*) = 1/6 so that *p*_II_(*n*, *m*) = 2/3 (except for the early game states with *n*≤2 and *m*≤2, where the first-mover advantage and win-probability is higher, owing to minor correction terms). The game-state advantage accrues most intensely when the player has substantially fewer characters than his opponent. Since we have shown this advantage for the moving player, this occurs in game states where *n* is substantially less than *m* (equivalently, where *r* is low).

## Concluding remarks

In this paper we have derived win probabilities under optimal strategy for play for two variants of the children’s game *Guess Who*, and we have derived corresponding results for the first-mover advantage and game-state advantage for the players. (Our analysis was derived under the assumption that the goal is to maximise the probability of winning the game.) We have also derived the set of pure optimal strategies for the rules variant where an incorrect character guess does not lead to loss of the game.

*Guess Who* is a type of ‘search race’ in which players seek to conclude their search successfully before their opponent does the same. The game using the variant rules is a discrete turn-based game, but its structure bears a resemblance to other forms of search race occurring in fields such as sporting competitions and industrial competitions. In particular, the game allows players to vary their strategy between “conservative” and “risky” strategies, and adapt this selection to the known state of progress of themselves and their opponent towards the goal. As with many search problems, the optimal strategy involves using a ‘split-in-half search’ to minimise the number of search steps. However, in game states where the other player is sufficiently close to winning it can be optimal to depart from this method and instead engage in ‘risky play’ which seeks to overtake the opponent in the search race.

The optimal strategy we have derived here is actually a set of pure strategies that are subgame perfect Nash equilibria of the relevant problem, and moreover, these form a single effective strategy set, insofar as all strategies in the set are best-responses to each other. We have also shown that under optimal play there is a first-mover advantage, such that first player has an advantage in the standard game of *Guess Who*. The probability of winning from any game state is easily calculated from the formulae in this paper, and has also been shown graphically in a heat map.

We conclude this analysis by summarising some basic facts about the optimal strategy and consequent win-probability in the two variants of *Guess Who*. Under optimal play there is an advantage to the first-mover, though the advantage is larger in the variant rules than in the official rules. If both players use an optimal strategy from the starting game state (23,23) then the probability that the first player will win the game is *p*_I_(23, 23) = 0.5595 under the official rules or *p*_II_(23, 23) = 0.6597 under the variant rules. Whilst the game is “unfair” it is possible to alter the starting game-state to obtain a fair game. There are a number of fair game states in the game where the waiting player has a game-state advantage that is of the same magnitude as the first-mover advantage of the moving player, leading to a fair game.

## Supporting information

S1 Appendix (DOCX)
