# Supplementary material for: Optimal guessing in ‘Guess Who’
Source: PLoS One. 2021 Mar 10;16(3):e0247361. doi: 10.1371/journal.pone.0247361 (PMC7946196; doi:10.1371/journal.pone.0247361)
Supplement: S1 Appendix — (DOCX) [file pone.0247361.s001.docx]

**S1 Appendix**

In this appendix we set out the proofs of the theorems in the main body of the paper. The proof of Theorems 1 and 3 are combined into a single proof, since they pertain to aspects of the same recursive optimisation problem. Our results make reference to two series of natural numbers that were mentioned in these theorems. By way of reminder these series are:

$$\begin{matrix} \left( \star k \right)_{k\mathbb{\in N}}=\left( 1, 2, 3,\underset{3\text{ times}}{\underbrace{6, \ldots, 6}},\underset{6\text{ times}}{\underbrace{12,\ldots, 12}},\underset{12\text{ times}}{\underbrace{24, \ldots, 24}},\underset{24\text{ times}}{\underbrace{48,\ldots, 48}}, \ldots\right)\text{,} \\ \left( k \right)_{k\mathbb{\in N}}=\left( \underset{3\text{ times}}{\underbrace{1, \ldots,1}},1, 2, 3, \underset{3\text{ times}}{\underbrace{3, \ldots,3}},4, 5, 6,\underset{6\text{ times}}{\underbrace{6,\ldots, 6}},7, 8, 9, 10, 11, 12,\underset{12\text{ times}}{\underbrace{12, \ldots, 12}}, 13,\ldots\right)\text{.} \end{matrix}$$

Both of these series can be given strict mathematical definitions, but these are not particularly illuminating, and we believe that the pattern is sufficiently clear so that no strict definition is needed. Before proceeding to prove the theorems in this paper we establish a useful identity involving the first series. We will prove the theorems in the paper slightly out of order since this is more useful than proceeding in the order they are presented in the body.

**Lemma 1:** For all $m\mathbb{\in N}$ have:

$$\sum_{k=1}^{m} \star k= \star m\left( m-\frac{\star m}{3} \right)+\frac{1}{3}\mathbb{\cdot I}\left( m\leq2 \right)\text{.}$$

**Proof of Lemma 1:** The result can easily be verified by substitution for small values of $m$. We will assume that this has been done for $m=1,2,3$ and we will provide the inductive step for values $m\geq4$. To facilitate this analysis, we denote the summation quantity as:

$$S\left( m \right)\equiv\sum_{k=1}^{m} \star k\text{.}$$

Our inductive step uses a proof by cases, using the fact that for all $m\geq4$ we have:

$$\star\left( m+1 \right)=\left\{ \begin{matrix} 2\star m & & \text{for }m= \star m\text{,} \\ \star m & & \text{for }m\neq\star m\text{.} \end{matrix} \right.$$

In the case first case we have:

$$S\left( m+1 \right)=S\left( m \right)+ \star\left( m+1 \right)$$

$$= \star m\left( m-\frac{\star m}{3} \right)+\frac{1}{3}\mathbb{\cdot I}\left( m\leq2 \right)+ \star\left( m+1 \right)$$

$$= \frac{\star\left( m+1 \right)}{2}\left( m-\frac{\star\left( m+1 \right)}{6} \right)+\frac{1}{3}\mathbb{\cdot I}\left( m\leq2 \right)+ \star\left( m+1 \right)$$

$$= \star\left( m+1 \right)\left( \frac{m}{2}-\frac{\star\left( m+1 \right)}{12} \right)+\frac{1}{3}\mathbb{\cdot I}\left( m\leq2 \right)+ \star\left( m+1 \right)$$

$$= \star\left( m+1 \right)\left( m-\frac{m}{2}-\frac{\star\left( m+1 \right)}{12} \right)+\frac{1}{3}\mathbb{\cdot I}\left( m\leq2 \right)+ \star\left( m+1 \right)$$

$$= \star\left( m+1 \right)\left( m-\frac{\star m}{2}-\frac{\star\left( m+1 \right)}{12} \right)+\frac{1}{3}\mathbb{\cdot I}\left( m\leq2 \right)+ \star\left( m+1 \right)$$

$$= \star\left( m+1 \right)\left( m-\frac{\star\left( m+1 \right)}{4}-\frac{\star\left( m+1 \right)}{12} \right)+\frac{1}{3}\mathbb{\cdot I}\left( m\leq2 \right)+ \star\left( m+1 \right)$$

$$= \star\left( m+1 \right)\left( m-\frac{\star\left( m+1 \right)}{3} \right)+\frac{1}{3}\mathbb{\cdot I}\left( m\leq2 \right)+ \star\left( m+1 \right)\text{.}$$

In the second case we have:

$$S\left( m+1 \right)=S\left( m \right)+ \star\left( m+1 \right)$$

$$= \star m\left( m-\frac{\star m}{3} \right)+\frac{1}{3}\mathbb{\cdot I}\left( m\leq2 \right)+ \star\left( m+1 \right)$$

$$= \star\left( m+1 \right)\left( m-\frac{\star\left( m+1 \right)}{3} \right)+\frac{1}{3}\mathbb{\cdot I}\left( m\leq2 \right)+ \star\left( m+1 \right)$$

$$= \star\left( m+1 \right)\left( \left( m+1 \right)-\frac{\star\left( m+1 \right)}{3} \right)+\frac{1}{3}\mathbb{\cdot I}\left( m\leq2 \right)\text{.}$$

In either case we have established the required inductive step, which completes the proof. ■

**Proof of Theorem 2:** We first consider the case where $\star n> \star m$. In this case $n> \star m$ so that $\min\left( n, \star k \right)= \star k$ for all $k\leq m$. This means that:

$$a_{\mathrm{II}}\left( n,m \right)=\sum_{k=1}^{m} \min\left( n, \star k \right)=\sum_{k=1}^{m} \star k= \star m\left( m-\frac{\star m}{3} \right)+\frac{1}{3}\mathbb{\cdot I}\left( m\leq2 \right)\text{.}$$

We now consider the case where $\star n\leq\star m$. If $n\geq4$ we have ${\star n}/2<n\leq\star m$ so that:

$$a_{\mathrm{II}}\left( n,m \right)=\sum_{k=1}^{m} \min\left( n, \star k \right)=\sum_{k=1}^{{\star n}/2} \min\left( n, \star k \right)+\sum_{k={\star n}/2+1}^{m} \min\left( n, \star k \right)$$

$$=\sum_{k=1}^{{\star n}/2} \star k+n\left( m-\frac{\star n}{2} \right)\text{.}$$

The first summation simplifies to:

$$\sum_{k=1}^{{\star n}/2} \star k=\left( \frac{\star n}{2}-\frac{\star n}{6} \right)\frac{\star n}{2}=\frac{\star n}{2}\left( 1-\frac{1}{3} \right)\frac{\star n}{2}=\frac{2}{3}\frac{\star n}{2}\frac{\star n}{2}=\frac{1}{6}\left( \star n \right)^{2}\text{.}$$

Hence, we have:

$$a_{\mathrm{II}}\left( n,m \right)=\frac{1}{6}\left( \star n \right)^{2}+n\left( m-\frac{\star n}{2} \right)=nm-\frac{\star n}{2}\left( n-\frac{\star n}{3} \right)\text{.}$$

The other cases for $n=1, 2, 3$ can easily be checked by substitution. ◼

**Proof of Theorems 1 and 3:** To prove Theorem 1 we need to show that the specified form for $a$ satisfies the baseline case $a_{\mathrm{II}}\left( 1,m \right)=m$ and the recursive equations:

$$\begin{matrix} a_{\mathrm{II}}\left( n,m \right)=\max_{1\leq s<n} a_{\mathrm{II}}\left( n,m | s \right)\text{,} \\ a_{\mathrm{II}}\left( n,m | s \right)=\left\{ \begin{matrix} nm-a_{\mathrm{II}}\left( m,n-s \right) & s=1\text{,} \\ nm-a_{\mathrm{II}}\left( m,s \right)-a_{\mathrm{II}}\left( m,n-s \right) & s>1\text{.} \end{matrix} \right. \end{matrix}$$

To prove Theorem 3 we need to show that the argument values maximising the first equation are as shown in that result. Both aspects of the problem are shown in the present proof. The key to obtaining the result is to set up an induction using an ordering of all possible game states. To cover all game-states we will proceed for each $k\mathbb{\in N}$ by obtaining $a_{\mathrm{II}}\left( k,m \right)$ for all $m\geq k$ and then obtaining $a_{\mathrm{II}}\left( n,k \right)$ for all $n\geq k$. This ordering allows us to obtain the win-probability recursively from the previous outcomes in the induction.

We will begin by verifying that the solution form is correct for values up to $k=3$. We have the baseline case $a_{\mathrm{II}}\left( 1,m \right)=m$ and we can then apply the recursive formula for $a_{\mathrm{II}}$ to obtain:

$$\begin{matrix} a_{\mathrm{II}}\left( 1,m \right)=m & & a_{\mathrm{II}}\left( n,1 \right)=1 & & n,m\geq1\text{,} \\ a_{\mathrm{II}}\left( 2,m \right)=2m-1 & & a_{\mathrm{II}}\left( n,2 \right)=3 & & n,m\geq2\text{,} \\ a_{\mathrm{II}}\left( 3,m \right)=3m-3 & & a_{\mathrm{II}}\left( n,3 \right)=6 & & n,m\geq3\text{.} \end{matrix}$$

It is easily verified that these results are consistent with the form proposed in the Theorem 1, so we are satisfied for values up to $k=3$. (The interested reader might wish to verify this from the recursive equation as an exercise.) We will now use an inductive step to show that the form satisfies the requirement for $k\geq4$ (i.e., for all game-states with $n\geq4$ and $m\geq4$).

To establish the inductive step we need to determine the optimal guess applying at each given game-state. To do this, let $\Delta$ be the difference operator with respect to the argument value $s$. Taking the first difference we have:

$$\Delta a_{\mathrm{II}}\left( n,m | s \right)=a_{\mathrm{II}}\left( n,m | s+1 \right)-a_{\mathrm{II}}\left( n,m | s \right)$$

$$=\min\left( m, \star\left( n-s \right) \right)-\min\left( m, \star\left( s+1 \right) \right)\mathbb{-I}\left( s=1 \right)\text{.}$$

Taking the second difference we have:

$$\Delta^{2}a_{\mathrm{II}}\left( n,m | s \right)=\Delta a_{\mathrm{II}}\left( n,m | s+1 \right)-\Delta a_{\mathrm{II}}\left( n,m | s \right)$$

$$=\min\left( m, \star\left( n-s-1 \right) \right)-\min\left( m, \star\left( n-s \right) \right)$$

$$+\min\left( m, \star\left( s+1 \right) \right)-\min\left( m, \star\left( s+2 \right) \right)\mathbb{-I}\left( s=1 \right)\text{.}$$

From this expression it is easy to see that $\Delta^{2}a_{\mathrm{II}}\left( n,m | s \right)\leq0$ so that the function $a_{\mathrm{II}}\left( n,m | s \right)$ is weakly concave with respect to the argument value $s$. This means that the maximising values for $s$ must be a connected set of integers with lower and upper bounds given respectively by:

$$\begin{matrix} \overline{s}_{*}\left( n,m \right)\equiv\max\left\{ s | \Delta a_{\mathrm{II}}\left( n,m | s+1 \right)>0 \right\}\text{,} \\ \overline{s}_{*}\left( n,m \right)\equiv\min\left\{ s | \Delta a_{\mathrm{II}}\left( n,m | s \right)<0 \right\}\text{.} \end{matrix}$$

In order to establish that the range in Theorem 3 is the correct solution we need to establish that these are the proper boundary values. We point out the different cases and leave it as sufficient to note the direction of the difference values in these cases.

**Lower Bounds:** If $\star n\leq\star m$ it can easily be shown that:

$$\begin{matrix} \Delta a_{\mathrm{II}}\left( n,m | s \right)>0 & & \text{for }s<n\text{,} \\ \Delta a_{\mathrm{II}}\left( n,m | s \right)\leq0 & & \text{for }s\geq n\text{.} \end{matrix}$$

If $\star n> \star m$ it can easily be shown that:

$$\begin{matrix} \Delta a_{\mathrm{II}}\left( n,m | s \right)>0 & & \text{for }s<\left( \star m \right)/2\text{,} \\ \Delta a_{\mathrm{II}}\left( n,m | s \right)\leq0 & & \text{for }s\geq\left( \star m \right)/2\text{.} \end{matrix}$$

This establishes that the lower bounds $\underline{s}_{*}\left( n,m \right)$ set out in Theorem 3 are maximising values, and no values below these are maximising values.

**Upper Bounds:** If $m\leq2$ or $n\leq3$ then it can easily be shown that $\Delta a_{\mathrm{II}}\left( n,m | 1 \right)<0$ so that $\overline{s}_{*}\left( n,m \right)=1$. If $m\geq3$ and $n=4$ it can easily be shown that:

$$\begin{matrix} \Delta a_{\mathrm{II}}\left( n,m | s \right)\geq0 & & \text{for }s<2\text{,} \\ \Delta a_{\mathrm{II}}\left( n,m | s \right)<0 & & \text{for }s\geq2\text{.} \end{matrix}$$

If $m\geq3$ and $n>4$ it can easily be shown that:

$$\begin{matrix} \Delta a_{\mathrm{II}}\left( n,m | s \right)\geq0 & & \text{for }s<n-\underline{s}_{*}\left( n,m \right)\text{,} \\ \Delta a_{\mathrm{II}}\left( n,m | s \right)<0 & & \text{for }s\geq n-\underline{s}_{*}\left( n,m \right)\text{.} \end{matrix}$$

This establishes that the upper bounds $\overline{s}_{*}\left( n,m \right)$ set out in Theorem 3 are maximising values, and no values above these are maximising values.

This establishes that Theorem 3 gives the full range of maximising values for the function $a_{\mathrm{II}}\left( n,m | s \right)$ which is the range of all optimal strategies. To establish Theorem 1 it remains only to show that the maximised value satisfies the requirements of the recursive equations. To do this it is sufficient to pick any one of the optimal strategies from Theorem 3 and substitute this to obtain the maximised value in the recursive equations; for simplicity we will use the particular optimal strategy in Corollary 1. For this strategy we have the ‘split-in-half search’ $s=\left\lfloor n/2 \right\rfloor$ on every guess with $n\geq4$ and $m\geq4$ (i.e., for all game-states we are currently considering). Since $\star\left\lfloor n/2 \right\rfloor= \star\left\lceil n/2 \right\rceil= {\star n}/2$ it follows from Theorem 2 that:

$$a_{\mathrm{II}}\left( m,\left\lfloor n/2 \right\rfloor\right)=\left\{ \begin{matrix} m\left\lfloor n/2 \right\rfloor-\frac{\star m}{2}\left( m-\frac{\star m}{3} \right) & & \text{if } \star m\leq{\star n}/2\text{,} \\ \frac{\star n}{2}\left( \left\lfloor n/2 \right\rfloor-\frac{\star n}{6} \right) & & \text{if } \star m>{\star n}/2\text{,} \end{matrix} \right.$$

and:

$$a_{\mathrm{II}}\left( m,\left\lceil n/2 \right\rceil\right)=\left\{ \begin{matrix} m\left\lceil n/2 \right\rceil-\frac{\star m}{2}\left( m-\frac{\star m}{3} \right) & & \text{if } \star m\leq{\star n}/2\text{,} \\ \frac{\star n}{2}\left( \left\lceil n/2 \right\rceil-\frac{\star n}{6} \right) & & \text{if } \star m>{\star n}/2\text{.} \end{matrix} \right.$$

Hence, we have:

$$a_{\mathrm{II}}\left( n,m | s \right)=nm-a_{\mathrm{II}}\left( m,s \right)-a_{\mathrm{II}}\left( m,n-s \right)$$

$$=nm-a_{\mathrm{II}}\left( m,\left\lfloor n/2 \right\rfloor\right)-a_{\mathrm{II}}\left( m,\left\lceil n/2 \right\rceil\right)$$

$$=\left\{ \begin{matrix} nm-m\left\lfloor n/2 \right\rfloor-m\left\lceil n/2 \right\rceil+ \star m\left( m-\frac{\star m}{3} \right) & & \text{if } \star m\leq{\star n}/2\text{,} \\ nm-\frac{\star n}{2}\left( \left\lfloor n/2 \right\rfloor-\frac{\star n}{6} \right)-\frac{\star n}{2}\left( \left\lceil n/2 \right\rceil-\frac{\star n}{6} \right) & & \text{if } \star m>{\star n}/2\text{.} \end{matrix} \right.$$

$$=\left\{ \begin{matrix} \star m\left( m-\frac{\star m}{3} \right) & & \text{if } \star m\leq{\star n}/2\text{,} \\ nm-\frac{\star n}{2}\left( 1-\frac{\star n}{3} \right) & & \text{if } \star m> {\star n}/2\text{.} \end{matrix} \right.$$

$$=\left\{ \begin{matrix} \star m\left( m-\frac{\star m}{3} \right) & & \text{if } \star n\geq2\star m\text{,} \\ nm-\frac{\star n}{2}\left( 1-\frac{\star n}{3} \right) & & \text{if } \star n<2\star m\text{.} \end{matrix} \right.$$

$$=\left\{ \begin{matrix} nm-\frac{\star n}{2}\left( n-\frac{\star n}{3} \right) & & \text{if } \star n\leq\star m\text{,} \\ \star m\left( m-\frac{\star m}{3} \right) & & \text{if } \star n> \star m\text{.} \end{matrix} \right.$$

This gives us:

$a_{s}\left( n,m \right)=a_{\mathrm{II}}\left( n,m \right)=\max_{1\leq s\leq n} a_{\mathrm{II}}\left( n,m | s \right)$.

This shows that the specified form of the win-probability in Theorem 1 is correct, and thereby completes the proof. ◼

|  |  | **Values of** $\boldsymbol{a}_{\mathbf{I}}\left( \boldsymbol{n,m} \right)\boldsymbol{=nm\cdot p}\left( \boldsymbol{n,m} \right)$ | | | | | | | | | | | | | | | | | | | | | | |
| --- | --- | --- | --- | --- | --- | --- | --- | --- | --- | --- | --- | --- | --- | --- | --- | --- | --- | --- | --- | --- | --- | --- | --- | --- |
|  |  | ***m*** | | | | | | | | | | | | | | | | | | | | | | |
|  |  | **1** | **2** | **3** | **4** | **5** | **6** | **7** | **8** | **9** | **10** | **11** | **12** | **13** | **14** | **15** | **16** | **17** | **18** | **19** | **20** | **21** | **22** | **23** |
| ***n*** | **1** | 1 | 2 | 3 | 4 | 5 | 6 | 7 | 8 | 9 | 10 | 11 | 12 | 13 | 14 | 15 | 16 | 17 | 18 | 19 | 20 | 21 | 22 | 23 |
|  | **2** | 1 | 2 | 4 | 6 | 8 | 10 | 12 | 14 | 16 | 18 | 20 | 22 | 24 | 26 | 28 | 30 | 32 | 34 | 36 | 38 | 40 | 42 | 44 |
|  | **3** | 1 | 3 | 5 | 7 | 10 | 13 | 16 | 19 | 22 | 25 | 28 | 31 | 34 | 37 | 40 | 43 | 46 | 49 | 52 | 55 | 58 | 61 | 64 |
|  | **4** | 1 | 4 | 6 | 9 | 12 | 16 | 20 | 24 | 28 | 32 | 36 | 40 | 44 | 48 | 52 | 56 | 60 | 64 | 68 | 72 | 76 | 80 | 84 |
|  | **5** | 1 | 4 | 7 | 10 | 14 | 18 | 22 | 26 | 31 | 36 | 41 | 46 | 51 | 56 | 61 | 66 | 71 | 76 | 81 | 86 | 91 | 96 | 101 |
|  | **6** | 1 | 4 | 8 | 12 | 16 | 20 | 25 | 30 | 35 | 40 | 46 | 52 | 58 | 64 | 70 | 76 | 82 | 88 | 94 | 100 | 106 | 112 | 118 |
|  | **7** | 1 | 4 | 9 | 13 | 18 | 22 | 27 | 32 | 38 | 44 | 51 | 58 | 65 | 72 | 79 | 86 | 93 | 100 | 107 | 114 | 121 | 128 | 135 |
|  | **8** | 1 | 4 | 10 | 14 | 20 | 24 | 30 | 36 | 42 | 48 | 56 | 64 | 72 | 80 | 88 | 96 | 104 | 112 | 120 | 128 | 136 | 144 | 152 |
|  | **9** | 1 | 4 | 10 | 15 | 21 | 26 | 32 | 38 | 45 | 52 | 60 | 68 | 76 | 84 | 92 | 100 | 109 | 118 | 127 | 136 | 145 | 154 | 163 |
|  | **10** | 1 | 4 | 10 | 16 | 22 | 28 | 35 | 42 | 49 | 56 | 64 | 72 | 81 | 90 | 99 | 108 | 117 | 126 | 135 | 144 | 154 | 164 | 174 |
|  | **11** | 1 | 4 | 10 | 16 | 23 | 30 | 37 | 44 | 52 | 60 | 68 | 76 | 85 | 94 | 103 | 112 | 122 | 132 | 142 | 152 | 163 | 174 | 185 |
|  | **12** | 1 | 4 | 10 | 16 | 24 | 32 | 40 | 48 | 56 | 64 | 72 | 80 | 90 | 100 | 110 | 120 | 130 | 140 | 150 | 160 | 172 | 184 | 196 |
|  | **13** | 1 | 4 | 10 | 16 | 25 | 33 | 42 | 50 | 59 | 67 | 76 | 84 | 94 | 104 | 114 | 124 | 135 | 146 | 157 | 168 | 181 | 194 | 207 |
|  | **14** | 1 | 4 | 10 | 16 | 26 | 34 | 44 | 52 | 62 | 70 | 80 | 88 | 99 | 110 | 121 | 132 | 143 | 154 | 165 | 176 | 190 | 204 | 218 |
|  | **15** | 1 | 4 | 10 | 16 | 27 | 35 | 46 | 54 | 65 | 73 | 84 | 92 | 103 | 114 | 125 | 136 | 148 | 160 | 172 | 184 | 199 | 214 | 229 |
|  | **16** | 1 | 4 | 10 | 16 | 28 | 36 | 48 | 56 | 68 | 76 | 88 | 96 | 108 | 120 | 132 | 144 | 156 | 168 | 180 | 192 | 208 | 224 | 240 |
|  | **17** | 1 | 4 | 10 | 16 | 28 | 37 | 49 | 58 | 70 | 79 | 91 | 100 | 112 | 124 | 136 | 148 | 161 | 174 | 187 | 200 | 216 | 232 | 248 |
|  | **18** | 1 | 4 | 10 | 16 | 28 | 38 | 50 | 60 | 72 | 82 | 94 | 104 | 117 | 130 | 143 | 156 | 169 | 182 | 195 | 208 | 224 | 240 | 256 |
|  | **19** | 1 | 4 | 10 | 16 | 28 | 39 | 51 | 62 | 74 | 85 | 97 | 108 | 121 | 134 | 147 | 160 | 174 | 188 | 202 | 216 | 232 | 248 | 264 |
|  | **20** | 1 | 4 | 10 | 16 | 28 | 40 | 52 | 64 | 76 | 88 | 100 | 112 | 126 | 140 | 154 | 168 | 182 | 196 | 210 | 224 | 240 | 256 | 272 |
|  | **21** | 1 | 4 | 10 | 16 | 28 | 40 | 52 | 64 | 77 | 90 | 103 | 116 | 130 | 144 | 158 | 172 | 187 | 202 | 217 | 232 | 248 | 264 | 280 |
|  | **22** | 1 | 4 | 10 | 16 | 28 | 40 | 52 | 64 | 78 | 92 | 106 | 120 | 135 | 150 | 165 | 180 | 195 | 210 | 225 | 240 | 256 | 272 | 288 |
|  | **23** | 1 | 4 | 10 | 16 | 28 | 40 | 52 | 64 | 79 | 94 | 109 | 124 | 139 | 154 | 169 | 184 | 200 | 216 | 232 | 248 | 264 | 280 | 296 |

|  |  | **Values of** $\boldsymbol{a}_{\mathbf{II}}\left( \boldsymbol{n,m} \right)\boldsymbol{=nm\cdot p}\left( \boldsymbol{n,m} \right)$ | | | | | | | | | | | | | | | | | | | | | | |
| --- | --- | --- | --- | --- | --- | --- | --- | --- | --- | --- | --- | --- | --- | --- | --- | --- | --- | --- | --- | --- | --- | --- | --- | --- |
|  |  | ***m*** | | | | | | | | | | | | | | | | | | | | | | |
|  |  | **1** | **2** | **3** | **4** | **5** | **6** | **7** | **8** | **9** | **10** | **11** | **12** | **13** | **14** | **15** | **16** | **17** | **18** | **19** | **20** | **21** | **22** | **23** |
| ***n*** | **1** | 1 | 2 | 3 | 4 | 5 | 6 | 7 | 8 | 9 | 10 | 11 | 12 | 13 | 14 | 15 | 16 | 17 | 18 | 19 | 20 | 21 | 22 | 23 |
|  | **2** | 1 | 3 | 5 | 7 | 9 | 11 | 13 | 15 | 17 | 19 | 21 | 23 | 25 | 27 | 29 | 31 | 33 | 35 | 37 | 39 | 41 | 43 | 45 |
|  | **3** | 1 | 3 | 6 | 9 | 12 | 15 | 18 | 21 | 24 | 27 | 30 | 33 | 36 | 39 | 42 | 45 | 48 | 51 | 54 | 57 | 60 | 63 | 66 |
|  | **4** | 1 | 3 | 6 | 10 | 14 | 18 | 22 | 26 | 30 | 34 | 38 | 42 | 46 | 50 | 54 | 58 | 62 | 66 | 70 | 74 | 78 | 82 | 86 |
|  | **5** | 1 | 3 | 6 | 11 | 16 | 21 | 26 | 31 | 36 | 41 | 46 | 51 | 56 | 61 | 66 | 71 | 76 | 81 | 86 | 91 | 96 | 101 | 106 |
|  | **6** | 1 | 3 | 6 | 12 | 18 | 24 | 30 | 36 | 42 | 48 | 54 | 60 | 66 | 72 | 78 | 84 | 90 | 96 | 102 | 108 | 114 | 120 | 126 |
|  | **7** | 1 | 3 | 6 | 12 | 18 | 24 | 31 | 38 | 45 | 52 | 59 | 66 | 73 | 80 | 87 | 94 | 101 | 108 | 115 | 122 | 129 | 136 | 143 |
|  | **8** | 1 | 3 | 6 | 12 | 18 | 24 | 32 | 40 | 48 | 56 | 64 | 72 | 80 | 88 | 96 | 104 | 112 | 120 | 128 | 136 | 144 | 152 | 160 |
|  | **9** | 1 | 3 | 6 | 12 | 18 | 24 | 33 | 42 | 51 | 60 | 69 | 78 | 87 | 96 | 105 | 114 | 123 | 132 | 141 | 150 | 159 | 168 | 177 |
|  | **10** | 1 | 3 | 6 | 12 | 18 | 24 | 34 | 44 | 54 | 64 | 74 | 84 | 94 | 104 | 114 | 124 | 134 | 144 | 154 | 164 | 174 | 184 | 194 |
|  | **11** | 1 | 3 | 6 | 12 | 18 | 24 | 35 | 46 | 57 | 68 | 79 | 90 | 101 | 112 | 123 | 134 | 145 | 156 | 167 | 178 | 189 | 200 | 211 |
|  | **12** | 1 | 3 | 6 | 12 | 18 | 24 | 36 | 48 | 60 | 72 | 84 | 96 | 108 | 120 | 132 | 144 | 156 | 168 | 180 | 192 | 204 | 216 | 228 |
|  | **13** | 1 | 3 | 6 | 12 | 18 | 24 | 36 | 48 | 60 | 72 | 84 | 96 | 109 | 122 | 135 | 148 | 161 | 174 | 187 | 200 | 213 | 226 | 239 |
|  | **14** | 1 | 3 | 6 | 12 | 18 | 24 | 36 | 48 | 60 | 72 | 84 | 96 | 110 | 124 | 138 | 152 | 166 | 180 | 194 | 208 | 222 | 236 | 250 |
|  | **15** | 1 | 3 | 6 | 12 | 18 | 24 | 36 | 48 | 60 | 72 | 84 | 96 | 111 | 126 | 141 | 156 | 171 | 186 | 201 | 216 | 231 | 246 | 261 |
|  | **16** | 1 | 3 | 6 | 12 | 18 | 24 | 36 | 48 | 60 | 72 | 84 | 96 | 112 | 128 | 144 | 160 | 176 | 192 | 208 | 224 | 240 | 256 | 272 |
|  | **17** | 1 | 3 | 6 | 12 | 18 | 24 | 36 | 48 | 60 | 72 | 84 | 96 | 113 | 130 | 147 | 164 | 181 | 198 | 215 | 232 | 249 | 266 | 283 |
|  | **18** | 1 | 3 | 6 | 12 | 18 | 24 | 36 | 48 | 60 | 72 | 84 | 96 | 114 | 132 | 150 | 168 | 186 | 204 | 222 | 240 | 258 | 276 | 294 |
|  | **19** | 1 | 3 | 6 | 12 | 18 | 24 | 36 | 48 | 60 | 72 | 84 | 96 | 115 | 134 | 153 | 172 | 191 | 210 | 229 | 248 | 267 | 286 | 305 |
|  | **20** | 1 | 3 | 6 | 12 | 18 | 24 | 36 | 48 | 60 | 72 | 84 | 96 | 116 | 136 | 156 | 176 | 196 | 216 | 236 | 256 | 276 | 296 | 316 |
|  | **21** | 1 | 3 | 6 | 12 | 18 | 24 | 36 | 48 | 60 | 72 | 84 | 96 | 117 | 138 | 159 | 180 | 201 | 222 | 243 | 264 | 285 | 306 | 327 |
|  | **22** | 1 | 3 | 6 | 12 | 18 | 24 | 36 | 48 | 60 | 72 | 84 | 96 | 118 | 140 | 162 | 184 | 206 | 228 | 250 | 272 | 294 | 316 | 338 |
|  | **23** | 1 | 3 | 6 | 12 | 18 | 24 | 36 | 48 | 60 | 72 | 84 | 96 | 119 | 142 | 165 | 188 | 211 | 234 | 257 | 280 | 303 | 326 | 349 |

|  |  | $\boldsymbol{n}>\boldsymbol{m}$ |  | $\boldsymbol{n\leq}\boldsymbol{m}$ |
| --- | --- | --- | --- | --- |

|  |  | **Optimal Strategy Range (variant rules) (Lower Bound)** | | | | | | | | | | | | | | | | | | | | | | |
| --- | --- | --- | --- | --- | --- | --- | --- | --- | --- | --- | --- | --- | --- | --- | --- | --- | --- | --- | --- | --- | --- | --- | --- | --- |
|  |  | ***m*** | | | | | | | | | | | | | | | | | | | | | | |
|  |  | **1** | **2** | **3** | **4** | **5** | **6** | **7** | **8** | **9** | **10** | **11** | **12** | **13** | **14** | **15** | **16** | **17** | **18** | **19** | **20** | **21** | **22** | **23** |
| ***n*** | **1** | 1 | 1 | 1 | 1 | 1 | 1 | 1 | 1 | 1 | 1 | 1 | 1 | 1 | 1 | 1 | 1 | 1 | 1 | 1 | 1 | 1 | 1 | 1 |
|  | **2** | 1 | 1 | 1 | 1 | 1 | 1 | 1 | 1 | 1 | 1 | 1 | 1 | 1 | 1 | 1 | 1 | 1 | 1 | 1 | 1 | 1 | 1 | 1 |
|  | **3** | 1 | 1 | 1 | 1 | 1 | 1 | 1 | 1 | 1 | 1 | 1 | 1 | 1 | 1 | 1 | 1 | 1 | 1 | 1 | 1 | 1 | 1 | 1 |
|  | **4** | 1 | 1 | 1 | 1 | 1 | 1 | 1 | 1 | 1 | 1 | 1 | 1 | 1 | 1 | 1 | 1 | 1 | 1 | 1 | 1 | 1 | 1 | 1 |
|  | **5** | 1 | 1 | 1 | 2 | 2 | 2 | 2 | 2 | 2 | 2 | 2 | 2 | 2 | 2 | 2 | 2 | 2 | 2 | 2 | 2 | 2 | 2 | 2 |
|  | **6** | 1 | 1 | 1 | 3 | 3 | 3 | 3 | 3 | 3 | 3 | 3 | 3 | 3 | 3 | 3 | 3 | 3 | 3 | 3 | 3 | 3 | 3 | 3 |
|  | **7** | 1 | 1 | 1 | 3 | 3 | 3 | 3 | 3 | 3 | 3 | 3 | 3 | 3 | 3 | 3 | 3 | 3 | 3 | 3 | 3 | 3 | 3 | 3 |
|  | **8** | 1 | 1 | 1 | 3 | 3 | 3 | 3 | 3 | 3 | 3 | 3 | 3 | 3 | 3 | 3 | 3 | 3 | 3 | 3 | 3 | 3 | 3 | 3 |
|  | **9** | 1 | 1 | 1 | 3 | 3 | 3 | 3 | 3 | 3 | 3 | 3 | 3 | 3 | 3 | 3 | 3 | 3 | 3 | 3 | 3 | 3 | 3 | 3 |
|  | **10** | 1 | 1 | 1 | 3 | 3 | 3 | 4 | 4 | 4 | 4 | 4 | 4 | 4 | 4 | 4 | 4 | 4 | 4 | 4 | 4 | 4 | 4 | 4 |
|  | **11** | 1 | 1 | 1 | 3 | 3 | 3 | 5 | 5 | 5 | 5 | 5 | 5 | 5 | 5 | 5 | 5 | 5 | 5 | 5 | 5 | 5 | 5 | 5 |
|  | **12** | 1 | 1 | 1 | 3 | 3 | 3 | 6 | 6 | 6 | 6 | 6 | 6 | 6 | 6 | 6 | 6 | 6 | 6 | 6 | 6 | 6 | 6 | 6 |
|  | **13** | 1 | 1 | 1 | 3 | 3 | 3 | 6 | 6 | 6 | 6 | 6 | 6 | 6 | 6 | 6 | 6 | 6 | 6 | 6 | 6 | 6 | 6 | 6 |
|  | **14** | 1 | 1 | 1 | 3 | 3 | 3 | 6 | 6 | 6 | 6 | 6 | 6 | 6 | 6 | 6 | 6 | 6 | 6 | 6 | 6 | 6 | 6 | 6 |
|  | **15** | 1 | 1 | 1 | 3 | 3 | 3 | 6 | 6 | 6 | 6 | 6 | 6 | 6 | 6 | 6 | 6 | 6 | 6 | 6 | 6 | 6 | 6 | 6 |
|  | **16** | 1 | 1 | 1 | 3 | 3 | 3 | 6 | 6 | 6 | 6 | 6 | 6 | 6 | 6 | 6 | 6 | 6 | 6 | 6 | 6 | 6 | 6 | 6 |
|  | **17** | 1 | 1 | 1 | 3 | 3 | 3 | 6 | 6 | 6 | 6 | 6 | 6 | 6 | 6 | 6 | 6 | 6 | 6 | 6 | 6 | 6 | 6 | 6 |
|  | **18** | 1 | 1 | 1 | 3 | 3 | 3 | 6 | 6 | 6 | 6 | 6 | 6 | 6 | 6 | 6 | 6 | 6 | 6 | 6 | 6 | 6 | 6 | 6 |
|  | **19** | 1 | 1 | 1 | 3 | 3 | 3 | 6 | 6 | 6 | 6 | 6 | 6 | 7 | 7 | 7 | 7 | 7 | 7 | 7 | 7 | 7 | 7 | 7 |
|  | **20** | 1 | 1 | 1 | 3 | 3 | 3 | 6 | 6 | 6 | 6 | 6 | 6 | 8 | 8 | 8 | 8 | 8 | 8 | 8 | 8 | 8 | 8 | 8 |
|  | **21** | 1 | 1 | 1 | 3 | 3 | 3 | 6 | 6 | 6 | 6 | 6 | 6 | 9 | 9 | 9 | 9 | 9 | 9 | 9 | 9 | 9 | 9 | 9 |
|  | **22** | 1 | 1 | 1 | 3 | 3 | 3 | 6 | 6 | 6 | 6 | 6 | 6 | 10 | 10 | 10 | 10 | 10 | 10 | 10 | 10 | 10 | 10 | 10 |
|  | **23** | 1 | 1 | 1 | 3 | 3 | 3 | 6 | 6 | 6 | 6 | 6 | 6 | 11 | 11 | 11 | 11 | 11 | 11 | 11 | 11 | 11 | 11 | 11 |

|  |  | $\boldsymbol{n}>\boldsymbol{m}$ |  | $\boldsymbol{n\leq}\boldsymbol{m}$ |
| --- | --- | --- | --- | --- |

|  |  | **Optimal Strategy Range (variant rules) (Upper Bound)** | | | | | | | | | | | | | | | | | | | | | | |
| --- | --- | --- | --- | --- | --- | --- | --- | --- | --- | --- | --- | --- | --- | --- | --- | --- | --- | --- | --- | --- | --- | --- | --- | --- |
|  |  | ***m*** | | | | | | | | | | | | | | | | | | | | | | |
|  |  | **1** | **2** | **3** | **4** | **5** | **6** | **7** | **8** | **9** | **10** | **11** | **12** | **13** | **14** | **15** | **16** | **17** | **18** | **19** | **20** | **21** | **22** | **23** |
| ***n*** | **1** | 1 | 1 | 1 | 1 | 1 | 1 | 1 | 1 | 1 | 1 | 1 | 1 | 1 | 1 | 1 | 1 | 1 | 1 | 1 | 1 | 1 | 1 | 1 |
|  | **2** | 1 | 1 | 1 | 1 | 1 | 1 | 1 | 1 | 1 | 1 | 1 | 1 | 1 | 1 | 1 | 1 | 1 | 1 | 1 | 1 | 1 | 1 | 1 |
|  | **3** | 1 | 1 | 1 | 1 | 1 | 1 | 1 | 1 | 1 | 1 | 1 | 1 | 1 | 1 | 1 | 1 | 1 | 1 | 1 | 1 | 1 | 1 | 1 |
|  | **4** | 1 | 1 | 2 | 2 | 2 | 2 | 2 | 2 | 2 | 2 | 2 | 2 | 2 | 2 | 2 | 2 | 2 | 2 | 2 | 2 | 2 | 2 | 2 |
|  | **5** | 1 | 1 | 3 | 3 | 3 | 3 | 3 | 3 | 3 | 3 | 3 | 3 | 3 | 3 | 3 | 3 | 3 | 3 | 3 | 3 | 3 | 3 | 3 |
|  | **6** | 1 | 1 | 4 | 3 | 3 | 3 | 3 | 3 | 3 | 3 | 3 | 3 | 3 | 3 | 3 | 3 | 3 | 3 | 3 | 3 | 3 | 3 | 3 |
|  | **7** | 1 | 1 | 5 | 4 | 4 | 4 | 4 | 4 | 4 | 4 | 4 | 4 | 4 | 4 | 4 | 4 | 4 | 4 | 4 | 4 | 4 | 4 | 4 |
|  | **8** | 1 | 1 | 6 | 5 | 5 | 5 | 5 | 5 | 5 | 5 | 5 | 5 | 5 | 5 | 5 | 5 | 5 | 5 | 5 | 5 | 5 | 5 | 5 |
|  | **9** | 1 | 1 | 7 | 6 | 6 | 6 | 6 | 6 | 6 | 6 | 6 | 6 | 6 | 6 | 6 | 6 | 6 | 6 | 6 | 6 | 6 | 6 | 6 |
|  | **10** | 1 | 1 | 8 | 7 | 7 | 7 | 6 | 6 | 6 | 6 | 6 | 6 | 6 | 6 | 6 | 6 | 6 | 6 | 6 | 6 | 6 | 6 | 6 |
|  | **11** | 1 | 1 | 9 | 8 | 8 | 8 | 6 | 6 | 6 | 6 | 6 | 6 | 6 | 6 | 6 | 6 | 6 | 6 | 6 | 6 | 6 | 6 | 6 |
|  | **12** | 1 | 1 | 10 | 9 | 9 | 9 | 6 | 6 | 6 | 6 | 6 | 6 | 6 | 6 | 6 | 6 | 6 | 6 | 6 | 6 | 6 | 6 | 6 |
|  | **13** | 1 | 1 | 11 | 10 | 10 | 10 | 7 | 7 | 7 | 7 | 7 | 7 | 7 | 7 | 7 | 7 | 7 | 7 | 7 | 7 | 7 | 7 | 7 |
|  | **14** | 1 | 1 | 12 | 11 | 11 | 11 | 8 | 8 | 8 | 8 | 8 | 8 | 8 | 8 | 8 | 8 | 8 | 8 | 8 | 8 | 8 | 8 | 8 |
|  | **15** | 1 | 1 | 13 | 12 | 12 | 12 | 9 | 9 | 9 | 9 | 9 | 9 | 9 | 9 | 9 | 9 | 9 | 9 | 9 | 9 | 9 | 9 | 9 |
|  | **16** | 1 | 1 | 14 | 13 | 13 | 13 | 10 | 10 | 10 | 10 | 10 | 10 | 10 | 10 | 10 | 10 | 10 | 10 | 10 | 10 | 10 | 10 | 10 |
|  | **17** | 1 | 1 | 15 | 14 | 14 | 14 | 11 | 11 | 11 | 11 | 11 | 11 | 11 | 11 | 11 | 11 | 11 | 11 | 11 | 11 | 11 | 11 | 11 |
|  | **18** | 1 | 1 | 16 | 15 | 15 | 15 | 12 | 12 | 12 | 12 | 12 | 12 | 12 | 12 | 12 | 12 | 12 | 12 | 12 | 12 | 12 | 12 | 12 |
|  | **19** | 1 | 1 | 17 | 16 | 16 | 16 | 13 | 13 | 13 | 13 | 13 | 13 | 12 | 12 | 12 | 12 | 12 | 12 | 12 | 12 | 12 | 12 | 12 |
|  | **20** | 1 | 1 | 18 | 17 | 17 | 17 | 14 | 14 | 14 | 14 | 14 | 14 | 12 | 12 | 12 | 12 | 12 | 12 | 12 | 12 | 12 | 12 | 12 |
|  | **21** | 1 | 1 | 19 | 18 | 18 | 18 | 15 | 15 | 15 | 15 | 15 | 15 | 12 | 12 | 12 | 12 | 12 | 12 | 12 | 12 | 12 | 12 | 12 |
|  | **22** | 1 | 1 | 20 | 19 | 19 | 19 | 16 | 16 | 16 | 16 | 16 | 16 | 12 | 12 | 12 | 12 | 12 | 12 | 12 | 12 | 12 | 12 | 12 |
|  | **23** | 1 | 1 | 21 | 20 | 20 | 20 | 17 | 17 | 17 | 17 | 17 | 17 | 12 | 12 | 12 | 12 | 12 | 12 | 12 | 12 | 12 | 12 | 12 |
